# Thermal-based early breast cancer detection using inception V3, inception V4 and modified inception MV4

**DOI:** 10.1007/s00521-021-06372-1

**Published:** 2021-08-07

**Authors:** Mohammed Abdulla Salim Al Husaini, Mohamed Hadi Habaebi, Teddy Surya Gunawan, Md Rafiqul Islam, Elfatih A. A. Elsheikh, F. M. Suliman

**Affiliations:** 1 IoT & Wireless Communication Protocols Lab, Department of Electrical Computer Engineering, International Islamic University, Selangor, Malaysia; 2 Department of Electrical Engineering, College of Engineering, King Khalid University, Abha, 61421 Saudi Arabia

**Keywords:** Breast cancer, Inception V3, Inception V4, Inception MV4, Deep convolutional neural network, Thermography

## Abstract

Breast cancer is one of the most significant causes of death for women around the world. Breast thermography supported by deep convolutional neural networks is expected to contribute significantly to early detection and facilitate treatment at an early stage. The goal of this study is to investigate the behavior of different recent deep learning methods for identifying breast disorders. To evaluate our proposal, we built classifiers based on deep convolutional neural networks modelling inception V3, inception V4, and a modified version of the latter called inception MV4. MV4 was introduced to maintain the computational cost across all layers by making the resultant number of features and the number of pixel positions equal. DMR database was used for these deep learning models in classifying thermal images of healthy and sick patients. A set of epochs 3–30 were used in conjunction with learning rates 1 × 10^–3^, 1 × 10^–4^ and 1 × 10^–5^, Minibatch 10 and different optimization methods. The training results showed that inception V4 and MV4 with color images, a learning rate of 1 × 10^–4^, and SGDM optimization method, reached very high accuracy, verified through several experimental repetitions. With grayscale images, inception V3 outperforms V4 and MV4 by a considerable accuracy margin, for any optimization methods. In fact, the inception V3 (grayscale) performance is almost comparable to inception V4 and MV4 (color) performance but only after 20–30 epochs. inception MV4 achieved 7% faster classification response time compared to V4. The use of MV4 model is found to contribute to saving energy consumed and fluidity in arithmetic operations for the graphic processor. The results also indicate that increasing the number of layers may not necessarily be useful in improving the performance.

## Introduction

Upon conducting the statistics by the World Health Organization in 2018, it became clear that there is a large increase in patients with breast cancer and late detection, which had a negative impact on human health. Death or amputation cases became more commonplace than before. Smart alternatives are the best solution to tackle these problems. With the development of thermal imaging techniques, researchers used thermal imaging with deep learning to detect breast cancer in early stages. Currently, research efforts are continuing to raise the efficiency of artificial intelligence models and their detection accuracy. In this paper, we offer the use of deep learning models for early stage breast cancer detection, which raises the efficiency of deep convolutional neural networks compared to previous studies.

The motivation of this study is to achieve high classification accuracy while reducing the run time of the code implementing the deep learning models given above. Inception V4 is a promising technique to detect early breast cancer disease as it overperforms inception -v3 with the addition of further layers. However, it is reported in the literature that the addition of further layers may not necessarily lead to considerable gains in accuracy levels [13, 22, 23]. Therefore, a modified version of inception -v4 (MV-4) is introduced, in an attempt, to reduce the number of layers used to speed up the run time while having a minimal effect on accuracy level gained.

Therefore, the contributions of this paper are given as follows:

1. The application of inception v4 pure architecture to the problem of the early detection of breast cancer disease utilizing grayscale and colored images.
2. The modification of inception v4 model (MV4) through the reduction of layers used to achieve faster speed. The computational cost is maintained across all layers by making the resultant number of features and the number of pixel positions be equal.
3. The performance analysis and evaluation of the proposed framework in terms of standard performance metrics used in the literature.
4. The verification of model robustness through the examination of 100% accuracy levels achieved through repeated experimentation and statistical analysis.
5. The discussion of the advantages and limitations of the proposed framework.

## Related works

In this section, the related works are presented. The researchers in [1] used dynamic and static database. They studied 42 patients and took 20 thermal images for each patient. They divided the experiment into three stages. First, they made 20 pictures for one patient and placed them in one array. Second, the mean for the 20 pictures was calculated. Then the first and last pictures’ mean was calculated. Third, the medium image was used to train the network. Two types of thermography (color, and grayscale) were used for the convolutional neural network. The accuracy ratios for static color and grey images were 98% and 95%, respectively, but the accuracy ratios for dynamic color and greyscale were 95% and 92%, respectively.

The researchers in [2] proposed four experiments to determine the best convolutional neural network (CNN). In the first experiment, they divided the database as follows. A 50% is used for training, 20% for evaluation and 30% for testing. They used CNN with different optimization methods, such as Adaptive Moment (ADAM), Root Mean Square Prop (RMSPROP) and Stochastic Gradient Descent (SGD), with SGD demonstrating the best performance. In the second experiment, they trained a different model of CNN with changing the upper layer with a flatten layer or global average pooling layer. Results showed that the simplest CNN models with Flatten layer have high accuracy. In the third experiment, the researchers developed a Bayesian optimization to optimize fifty models of CNN and to obtain the highest value in F1 score. Finally, the fourth experiment used CNN models that obtained the highest value of F1 and modified the division of database as 80% for training and 20% for validation. The results indicated that deep CNN models, such as ResNet50, SeResNet50 and inception, have accuracies higher than 92%.

The researchers in [3] used a group of convolutional neural network models (AlexNet, GoogLeNet, ResNet-50, ResNet-101, inception-v3, VGG-16 and VGG-19). They divided the database into 70% for training and 30% for validation from a total of 173 thermal images—the learning rate set to five epochs. The results showed that the convolutional neural network VGG 16 has the highest percentage of accuracy of 91.18%, while no result shown for the convolutional neural network inception v3. The researchers in [4] suggested using the convolutional neural network consisting of two stages. The first one was for tagging and the second was for segmentation. In the first stage, the convolutional neural network model was Resnet-50 architecture, but in the second stage, the convolutional neural network model was V-Net. Learning rate was set to $0.5\times {10}^{-3}$ and the epoch was set to 50 while ADAM Optimization was used. They took thermography breast cancer from different angles (e.g., front view, right side, and left side). The result showed the accuracy can reach 91%.

In [5], researchers used four convolutional neural network models. These were ResNet101, DenseNet, MobileNetV2 and ShuffleNetV2. Besides, they used Database for Mastology Research (DMR) for thermal images and divided it into 80% for training and 20% for validation. Each model was set as follows: learning rate is 0.001, WeightLearnRateFactor is 10, BiasLearnRateFactor is 10 and the SGD optimization was used. Then, each model is trained by changing epoch value to 10, 20 and 30. Researchers reported that ResNet101 and DenseNet had achieved 100% accuracy within ten epochs.

Researcher in [6] converted the thermal images from DMR to grayscale, with the gray level per pixel is 256 was reduced. The gray level was reduced 32 times and the new gray level is stored in “Gray Level” register. A convolutional neural network with support vector machine (SVM) was used, and five features were used based on high classification accuracy, and relatively simple hardware implementation. The best five features selected from a total of 32 features using Gray level co-occurrence matrix (GLCM) and run-length matrix (RLM) were used. Finally, it has been implemented on an FPGA card for initial verification in the clinical environment. Results showed that the accuracy rate reached 90.06%.

The researchers in [7] suggested using the deep convolutional neural network (CNN) model inception V3. They used the DMR database with 1062 thermal images that were divided into 80% for training and 20% for validation. They added support vector machine at the end of the CNN model for classification and set the learning rate to 0.0001 and epoch to 15. The researchers did not mention the accuracy rate, but the percentage of the presence of the disease in the breast was reported.

The researchers in [8] used the convolutional and deconvolutional neural networks (C-DCNN) model. The researchers used two experiments to divide the breast area into a group of samples. For the first experiment, the total number of thermal images was 165, and they divided them into 132 images for training, 33 images for validation, while the IoU was set to 0.9424. In the second experiment, they used 150 thermal images for training, 10 images for validation, and IoU was set to 0.8340. They also set both experiments for the epoch of 1000 and a learning rate of 0.0001. Additionally, they used Otsu thresholding to change color images to greyscale before inserting them into the CDCNN while the graphic Hardware (GPU) model GTX1080Ti was used. The result showed that the CDCNN model achieved an accuracy level of 94%.

In another study [18], deep convolutional neural networks used Resnet18, Resnet34 and resnet50, resnet152, vgg16 and vgg19. They used thermal image database with 400 healthy and 400 patients and used 80% for training and 20% for verification. In addition to controlling the amount of learning with different values, Stochastic Gradient Descent Momentum (SGDM) optimization was used to compare the predictive accuracy of different CNN architectures to detect breast cancer in early stages. Finally, best results were obtained with resnet34 and resnet50, reaching a predictive accuracy of 100% in blind validation. In [19], various models of deep convolutional neural networks were used for semantic segmentation starting from naive patch-based classifiers to the more sophisticated encoder-decoder architecture for detecting hotspots in the thermal image. VGGNet, CascadeCNN, U-net, V-net and End-to-end CNN were used with settings of DCNN for a learning rate of 1e-4, ADAM optimization method, and total of 1200 thermal images. Moreover, Keras program was used with NVIDIA GeForce GTX TITAN X (12 GB) GPU for the simulations. The results obtained from CascadeCNN had the best training accuracy of 0.92, corresponding to the best validation accuracy of 0.87.

In [20], authors compared convolutional neural network with Bayes algorithm optimization (CNN + Bayes) to convolutional neural network (CNN). In addition, a total of 558 benign and 558 malignant thermographic images were used to train and validate convolutional neural network. The results of the convolutional neural network with Bayes obtained high accuracy of 98.95%. On the other hand, authors of [25] used a total of 1140 thermal images from PROENG database. They evaluated augmentation algorithms with rotation range = 5, shear range = 0.03, zoom range = 0.03, and horizontal flip = True rotation. They utilized inception v3 with setting to ‘ADAM’ optimization method and a learning rate of 1e − 4. Moreover, they used GPU with 16 GB of RAM, NVIDIA Tesla K80 and python software. Then set inception v3 in classification unit to (Global Average Pooling, Full Connected layer (512), Dropout (0.5), SoftMax. Besides, they kept last convolution layer unfrozen. Then, adjustment on learn rate, drop rate, and batch normalization were applied. The results obtained for inception V3 model are an accuracy level of 98.5%, 100% precision, 97.5% recall and 98.7% F1-score.

## Methodology

In this section, the general framework of the proposed methods to detect breast cancer using deep convolutional neural network and thermal images is presented in detail.

### Framework

**Fig. 1:**
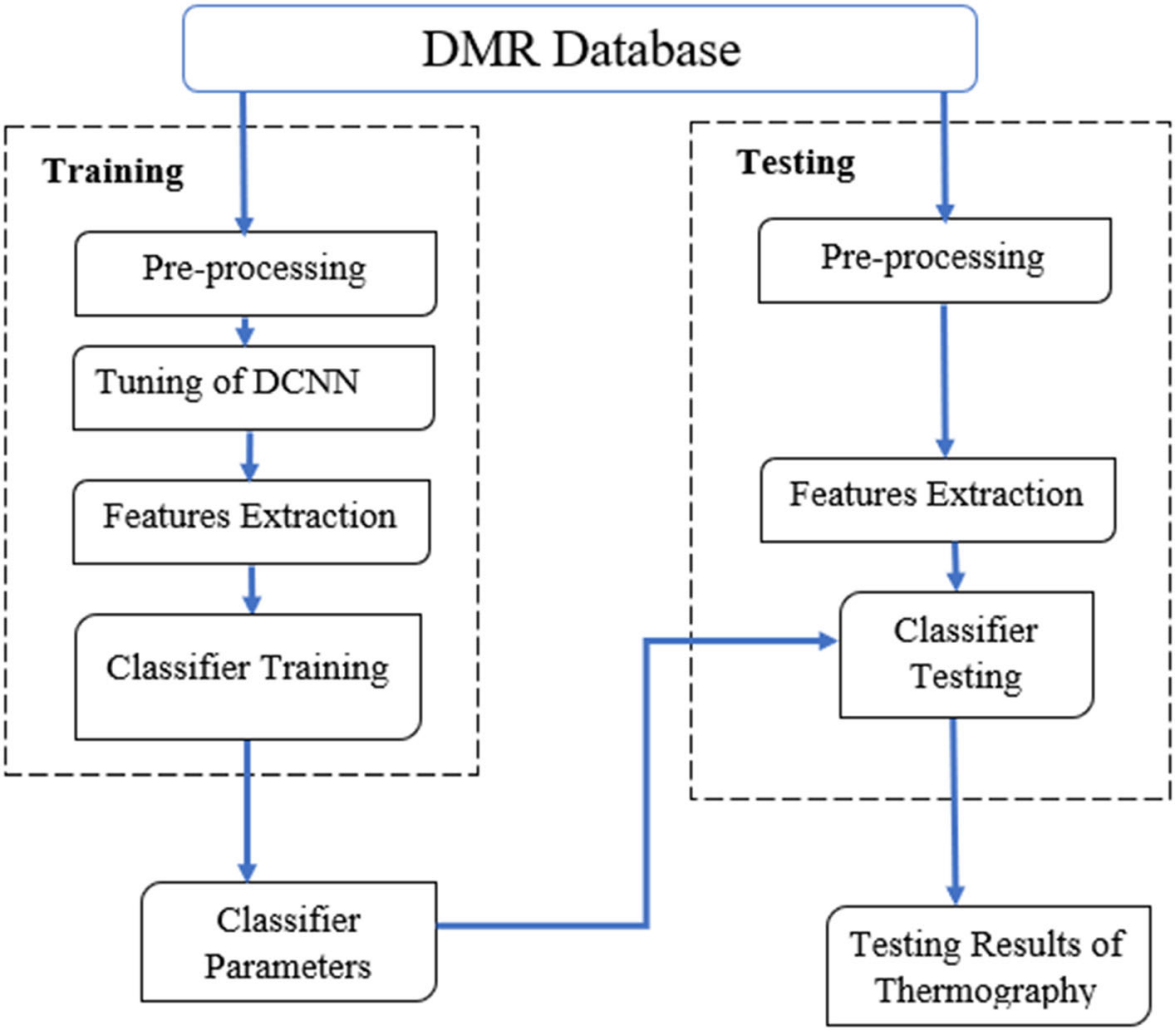
Flowchart of breast cancer detection process

The following box illustrates our approach to detecting breast cancer using the deep convolutional neural network. We will summarize the main operations as follows. First, two types of deep convolutional neural network are used, inception V3 and inception V4 to achieve the best accuracy in detecting breast cancer from thermal images. Second, a modified version of the deep convolutional neural network model inception V4 (MV4) is introduced (Fig. 1).

### Inception V3

Inception V3 model is an additional development design of useful CNN from Google that has obtained an accuracy of 0.928, top 5 at ILSVRC-2015 [26]. Inception had started by approximating a sparse structure (e.g., increasing depth and width of the network) and by clustering the similar non-uniform sparse data structure nodes into a dense structure, to achieve higher accuracy without straining the computational budget (more details can be found in [26]).

**Fig. 2:**
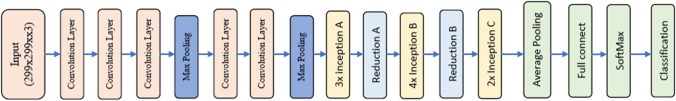
Inception V3 Model

The structure of the deep convolutional neural network inception V3 is shown in Fig. 2. The basic principles of structure of the inception V3 construction were formulated as follows:

1. A big number of signals are located close to each other. This can be utilized to create minimal convolutions. Neighboring signals are often correlated, and that means it is feasible to shrink the dimensions before applying the convolution without loss of data.
2. When increasing the free weight of resources for their successful utilization, it is required to increase the depth and width of the neural network at the same time.
3. It is inactive to utilize layers that sharply minimize the values of parameters, especially at the first of a convolutional neural network.
4. “Wide” layers learn very fast, which is important at big levels.

### Inception V4

**Fig. 3:**
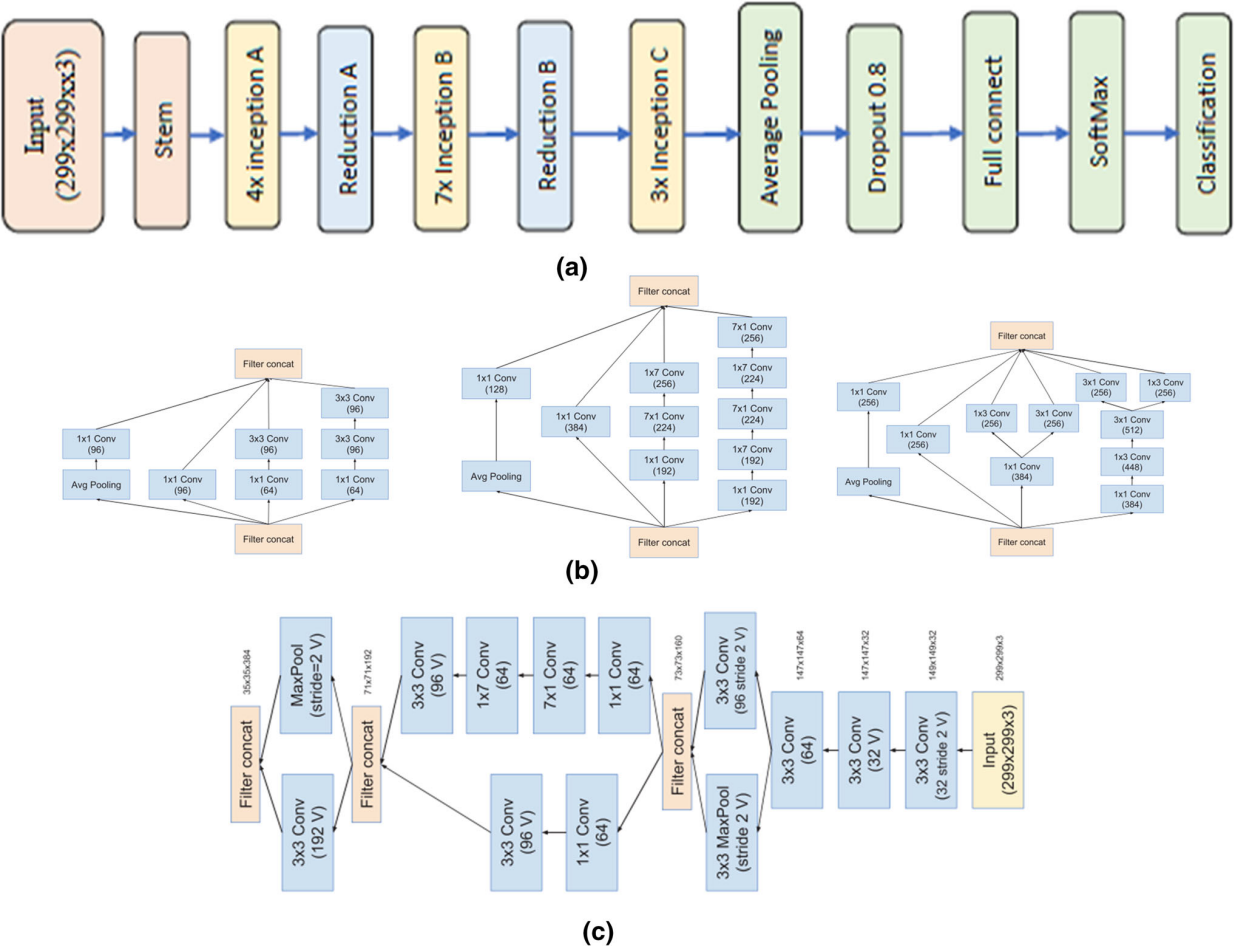
**a** Inception V4 Model, **b** Details of inception A, B and C layers, **c** Stem composition [16]

Inception-v4 is a deep convolutional neural network structure that builds on preceding iterations of the inception family by simplifying the architecture, adding stem layer and using more inception modules than inception-v3 with more uniform simplified architecture. The full configuration of the inception-v4 network is summarized in Fig. 3, which contains the overall planner and stem configuration, 4 inception A, 7 inception B and 3 inception C layers, as shown in Fig. 3 ((more details can be found in [16]).

### Proposed modified inception MV4

**Fig. 4:**
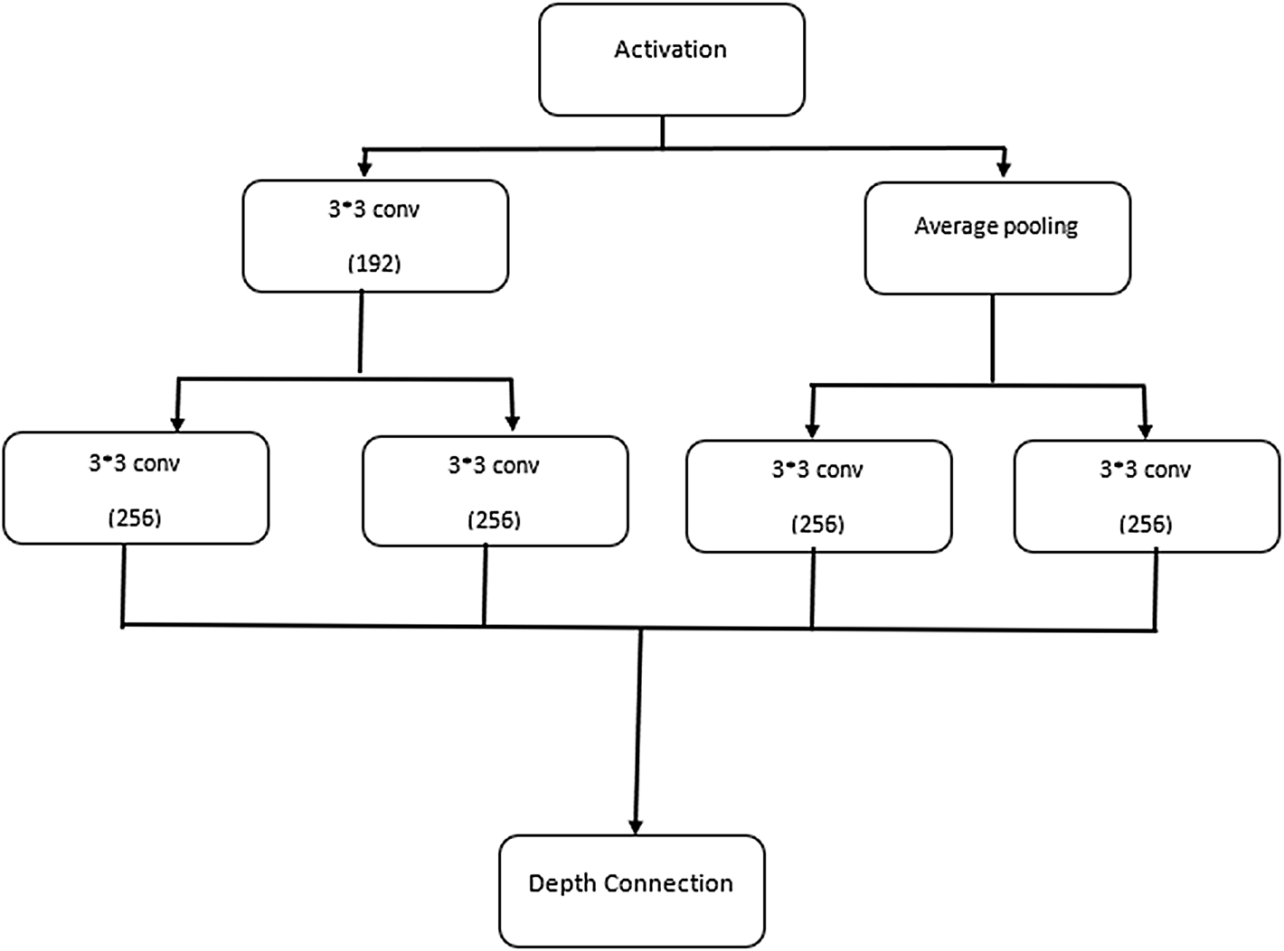
Modified inception B

A comparison was made between the network part inception B in inception V4 and the new network part inception B in MV4, as shown in Fig. 4. The adjustments made to inception B only, and these are as follows:

1. Added one convolutional layer under average pooling layer with size 3*3 and 256 filters. The number of filters has changed from 128 to 256 filters to preserve the number of extracted features.
2. Added two convolutional layers in parallel under the layer with 192 filters, each of them is having a filter size of 3*3, and 256 filters.
3. Removed the rest of the layers in inception B to preserve the extracted features with a total of 1,024 features.
4. Inception B contains 7 groups with multiple layers. All the 7 groups are modified by same settings above.

To verify the output results, we use the following formula:

Output layer size

1 $$=\frac{W-F+2P}{S}+1$$

W = input size.

F = filter size.

P = padding sitting.

S = stride setting.

In inception V3 and V4 models, both types of thermal images (color and grayscale) were used. In the training stages, different types of optimization algorithms were used, such as SGDM, ADAM and RMSPROP. Further three values of learning rates that were used in the experiments as recommended in the literature, e.g., ${1\times 10}^{-3}$ [9, 10] ${1\times 10}^{-4}$ and ${5\times 10}^{-4}$ [4]. Finally, training the network at different stages continued until the rapid response and accuracy of the deep convolutional neural network for the two proposed models are evident [11–13].

### Database

The DMR database used in this study consists of thermal images of 287 people, 48 of whom are sick, while the rest are healthy. FLIR SC 620 thermal camera was used in acquiring these images, that is characterized by its high resolution of 480*640 pixels, its excellent sensitivity of 0.04 and capture temperature between − 40 °C and 500 °C. The DMR data are characterized by its quality, where a protocol was developed to capture thermal images in patients. Strict protocols were followed while taking these thermal images, including subjects were not allowed to consume alcohol and caffeine, and not carrying out any physical activities or applying any creams or oils to the body. The room temperature was set between 18 °C and 25 °C. In addition, the cold air flow has been placed on the breast area until it met the required standard for taking pictures. Moreover, the thermal camera with a distance of one meter was placed in front of the patient and twenty pictures were taken from the front, one from the right side at 90° and another from the left side at 90° [14].

### Pre-processing

Pre-processing is extremely important for thermal images before inserting them into the deep convolutional neural network. Through it, automatic removal of unwanted areas, such as the armpits, hands, neck, and abdomen area, is done. Besides, it is required, before feeding a thermal image into different DCNN, to adjust suitably the size of the image.

### Features extraction

The extraction of features from thermal images is one of the basics of image analysis, which contributes to the accurate investigation of breast cancer in patients. Automated extracting features in the images using MATLAB depends on training Database and type of DCNN. A typical DCNN, such as inception V3 and V4, extracts a feature maps and recognition tasks without prior knowledge of the field-specific image feature. The typical layers in deep convolutional neural networks are the convolution layers and pooling layers. Convolution layers with kernel filters convolve input feature maps, followed by optional nonlinear function to produce output feature maps. The pooling layer takes only one value from the input map, forcing the resolution of the feature maps to decrease, to make the output feature maps stay invariant with respect to local deformations. Once features are extracted from convolution layers and pooling layers, with max-pooling and average pooling operations, fully connected layers mix the output features into a full feature vector. The output layer is the fully connected layer with the classification as the output result, allowing for automatic feature extraction and classification procedures together [17].

### Performance evaluation

We use a set of specialized equations to evaluate the experiments on the detection of breast cancer, including accuracy, sensitivity, specificity, positive predictive value, negative predictive value, false-positive rate, false-negative rate, likelihood ratio positive, likelihood ratio negative, an area under curve, Equal Error rate and F1 score[15]. These equations are calculated as follows:

2 $$\rho =\frac{TP}{TP+FP}$$

3 $$Accuracy=\frac{TP+TN}{TP+FP+FN+TN}$$

4 $$Specificity=\frac{TN}{FP+TN}$$

5 $$Sensitivity=\frac{TP}{TP+FN}$$

6 $$NPV=\frac{TN}{TN+FN}$$

7 $$FPR=\frac{FP}{TN+FP}$$

8 $$FNR=\frac{FN}{TP+FN}$$

9 $$LRP=\frac{Sensitivity}{1-Specificity}$$

10 $$LRP=\frac{1-Sensitivity}{Specificity}$$

11 $$AUC=\frac{Sensitivity+Specificity}{2}$$

12 $$EER=1-AUC$$

13 $$F1=2\frac{\left(Sensitivity \times \rho \right)}{\left(Sensitivity+\rho \right)}$$

## Experimental setups

Architecture of deep convolutional neural network inception V4 consists of seven stages: Steam, four inception A, Reduction A, seven inception B, Reduction B, three inception C and Classification [13]. In each of these stages, there is a group of convolutional layers and filters, as shown in Fig. 3. The weights in the Stem are set to zero to speed up the training process, using freezeWeights in MATLAB version 2020.

DCNN inception V3 model consists of three inception A, reduction A, four inception B, Reduction B, two inception C, and a classification layer. All these layers have 3*3 size filters with different numbers of filters, such as 32, 64, 96, 192, 256, and 384. Furthermore, there are two layers, ReLu activation and batch normalization layers, connected after each convolutional layer. Additionally, the deep convolutional neural network, Inspection V3, is available on the MATLAB Toolbox (Table 1).

**Table 1:** Parameters meaning

Symbol	Meaning
*TP*	True positive
*TN*	True negative
*FP*	False positive
*FN*	False negative
*Accu*	Accuracy
*Sen*	Sensitivity
*Spe*	Specificity
*P*	Precision
*NPV*	Negative predictive value
*FPR*	False-positive rate
*FNR*	False-negative rate
*LRP*	Likelihood ratio positive
*LRN*	Likelihood ratio negative
*AUC*	Area under curve
*EER* *F1*	Equal error rate Harmonic mean of precision and recall

Both DCNN inception V3 and inception V4 models require image sizes of 299*299 pixels. However, images in the DMR database may be varied in size, so the size of the images was adjusted automatically. Furthermore, additional augmentation operations were performed on the training images to increase the training data. For example, flip the training images randomly along the vertical axis and translate them up to 30 pixels randomly and scale them up to 10% both horizontally and vertically. This helps to preserve the exact details of the training images and prevents the network from overfitting.

In our experiments, we used a Dell computer 32 RAM Core i7 Hard disc 1 TB, and an added graphic card model Asus GeForce GTX 1660 memory 6 GB with MATLAB. The thermal image database of the breast cancer was downloaded from the website http://visual.ic.uff.br/en/proeng/. In our experiments, RGB-color images were used. In addition, we used 1019 healthy thermal images and 862 thermal images of breast cancer patients. Furthermore, since DMR database were utilized which were taken by the thermal camera FLIR SC 620, the image resolution was 480*640 pixel for 16-bit thermal images. To evaluate the performance of the deep convolutional neural network inception V3 and inception V4 models, four experiments were devised, as follows. In the first experiment, inception V3 was used with color images, while in the second experiment, it was grayscale images. In the third experiment, inception v4 was used with color images while it was used with grayscale images in the fourth experiment. All these four experiments were made with color image and grayscale with different values of epochs, learning rates and optimization methods.

The fifth experiment was designed to evaluate the performance of the inception MV4. The inception V4 model originally consisted of 226 layers and 280 connections while the Modified inception MV4 model consisted of 191 layers and 245 connections. The experimental parameters were different values of epochs (3, 4, 5, 6, 7, 8, 9, 10, 20, and 30), a learning rate of $1\times {10}^{-4}$, batch normalization with standard value to 10, different optimizations algorithm (SGDM, ADAM and RMSPROP) and color thermal images.

## Results

Table 2 shows the DCNN inception V3, trained using color images, has achieved accuracy ratio of 100% with the training period lasting 12.2 min, when the epoch was set to 6, the learning rate was $1\times {10}^{-3}$ , and with SGDM optimization. While the 100% accuracy was achieved with the ADAM Optimization with 7 epochs, $1\times {10}^{-4}$ learning rate, while the training time was 20.42 min. The RMSPROP Optimization achieved the 100% accuracy within 6 epochs and $1\times {10}^{-3}$ learning rate, but the time taken for training was 14.87 min.

**Table 2:** Detection accuracy of inception V3 by using color image

Inception V3 (Color)									
Epoch	SGDM			SGDM			RMSPROP		
	0.001	0.0001	0.0005	0.001	0.0001	0.0005	0.001	0.0001	0.0005
4	96.8	81.85	93.77	99.11	97.69	97.15	50.36	96.62	95.02
5	94.31	82.56	94.13	99.82	99.11	97.51	91.99	96.98	97.86
6	100	86.3	96.44	97.15	98.75	98.58	100	99.64	99.82
7	96.8	88.97	93.95	98.4	100	97.33	54.09	100	100
8	99.11	88.79	98.58	98.58	96.26	92.7	56.58	100	99.82
9	94.84	87.19	96.98	99.47	99.82	99.64	55.69	98.75	98.93
10	99.11	90.04	94.13	99.82	100	99.11	54.27	94.66	100
20	99.83	90.21	96.6	94.66	99.82	100	99.64	98.93	99.82
30	98.93	94.84	98.75	100	100	100	74.38	99.82	99.82

On the other hand, Table 3 displays the results of converting color images to grayscale and inserting them into DCNN inception V3. The results indicated that the best accuracy achieved was 99.82% for SGDM optimization when the epoch was 20 and learning rate $1\times {10}^{-3}$ , while the training time was 44.73 min. With ADAM optimization, the best accuracy of 100% were achieved when the epoch was 9, the learning rate was $1\times {10}^{-4}$ and the time taken by the network to train was 28.28 min. In addition, the RMSPROP shows accuracy of 100% also when the value of the epoch was 9, and the learning rate was $5\times {10}^{-4}$ , the time taken for training was 23.38 min. In summary, the results of the trained DCNN inception V3 to detect breast cancer indicate that the use of color images with SGDM optimization, produces highest accuracy level with less response time and less rounds of training.

**Table 3:** Detection accuracy of inception V3 by using grayscale image

Inception V3 (Grayscale)									
Epoch	SGDM			ADAM			RMSPROP		
	0.001	0.0001	0.0005	0.001	0.0001	0.0005	0.001	0.0001	0.0005
4	97.15	95.02	98.58	83.27	97.33	94.13	54.09	99.64	99.11
5	99.64	95.37	97.51	98.75	86.48	99.47	54.09	90.93	96.26
6	98.75	96.98	99.11	95.73	93.77	99.47	54.09	95.02	95.37
7	98.58	98.22	95.91	91.28	92.88	94.66	54.09	95.02	99.64
8	98.4	95.73	98.75	97.15	92.35	98.75	54.09	95.55	99.64
9	97.15	95.58	99.29	65.48	100	99.29	54.09	92.88	100
10	97.51	94.66	96.09	95.91	93.42	95.73	54.09	91.1	94.84
20	99.82	98.75	99.29	98.93	97.33	99.82	70.46	100	100
30	98.93	97.86	99.64	80.25	99.47	95.91	70.28	100	100

**Fig. 5:**
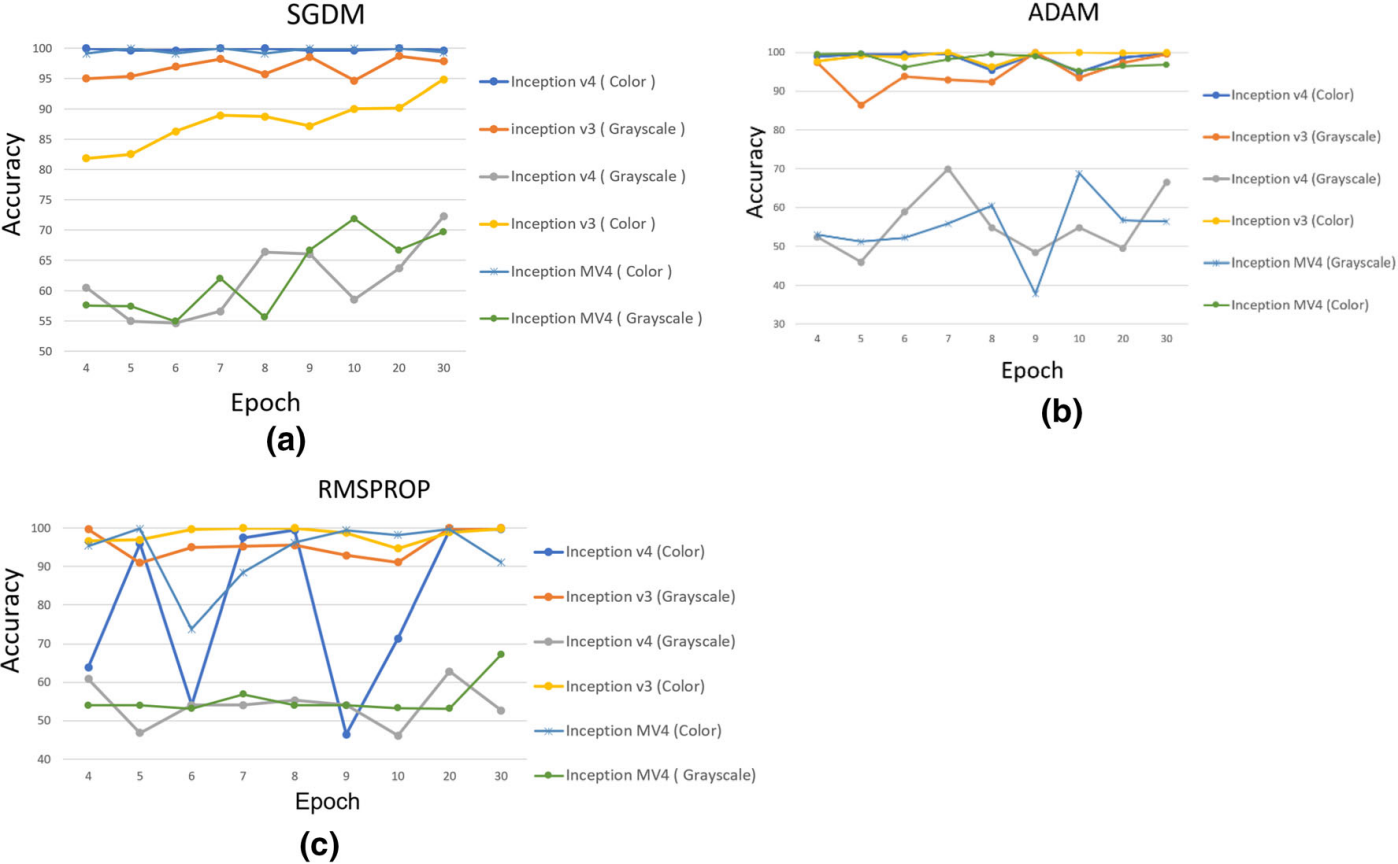
Detection accuracy of inception V3, V4 & MV4 by using Color and Grayscale image in: (**a**) SGDM optimization, (**b**) ADAM optimization, (**c**) RMSPROP optimization

As for the second part of the experiment in which we used DCNN inception V4, the results in Table 4 indicate its high performance, especially in the treatment of color thermal images. The inception V4 model achieved a 100% accuracy, Area Under Curve is 1 (see Table 6) by epoch 4 only, with a learning rate of $1\times {10}^{-4}$, and SGDM optimization (see Fig. 5a). As for RMSPROP and ADAM optimizations, inception V4 model did not reach the 100% accuracy level in all training stages. The training stages included 54 trials in different modes, but they managed to achieve the highest accuracy levels of 99.64% and 95.91%, respectively (see Fig. 4b, c).

**Table 4:** Detection accuracy of inception V4 by using color image

Inception V4 (Color)									
Epoch	SGDM			ADAM			RMSPROP		
	0.001	0.0001	0.0005	0.001	0.0001	0.0005	0.001	0.0001	0.0005
4	94.84	100	99.47	60.32	98.93	54.09	44.66	63.88	54.09
5	99.29	99.64	99.47	46.44	99.47	54.09	45.91	95.91	45.91
6	99.64	99.64	99.82	42.17	99.47	45.91	54.09	54.09	46.44
7	50.53	100	99.82	54.09	99.64	45.91	66.01	97.51	54.09
8	84.52	100	97.86	54.09	95.37	59.96	50.36	99.47	45.91
9	49.11	99.64	99.82	52.85	99.64	54.09	45.91	46.44	61.57
10	49.82	99.64	99.82	53.2	94.84	54.09	35.94	71.35	45.91
20	45.91	100	99.82	54.09	98.58	55.52	54.09	99.47	50
30	93.95	99.64	93.95	46.98	99.64	58.54	54.09	99.64	95.91

**Fig. 6:**
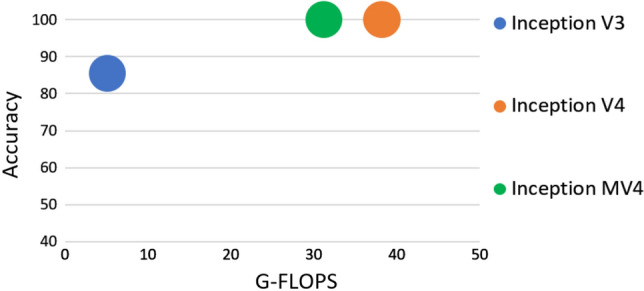
Giga floating-point operations per second (G-FLOPS) of inception V3, V4 & MV4

**Fig. 7:**
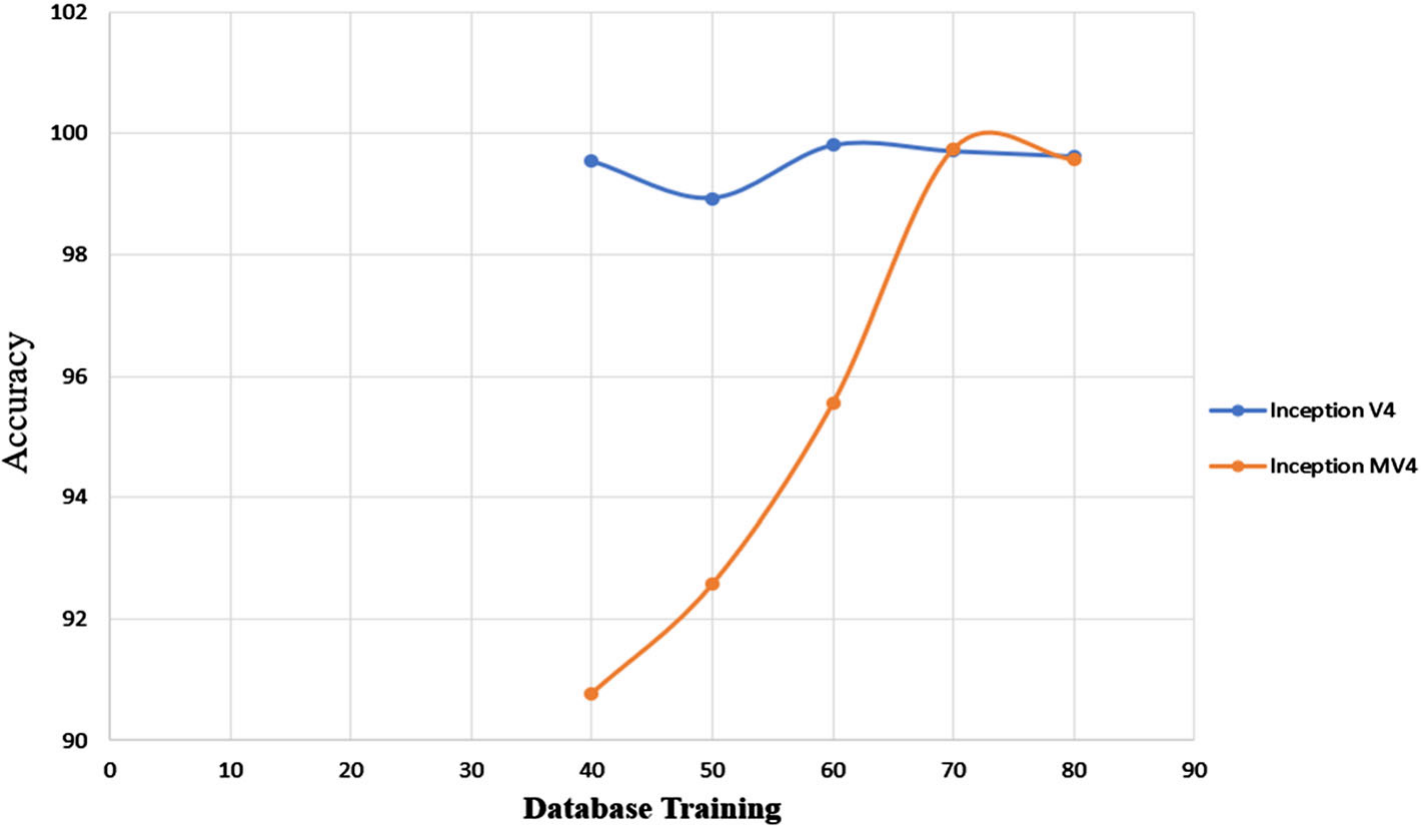
Average accuracy of different database training and testing for inception V4 and MV4

**Fig. 8:**
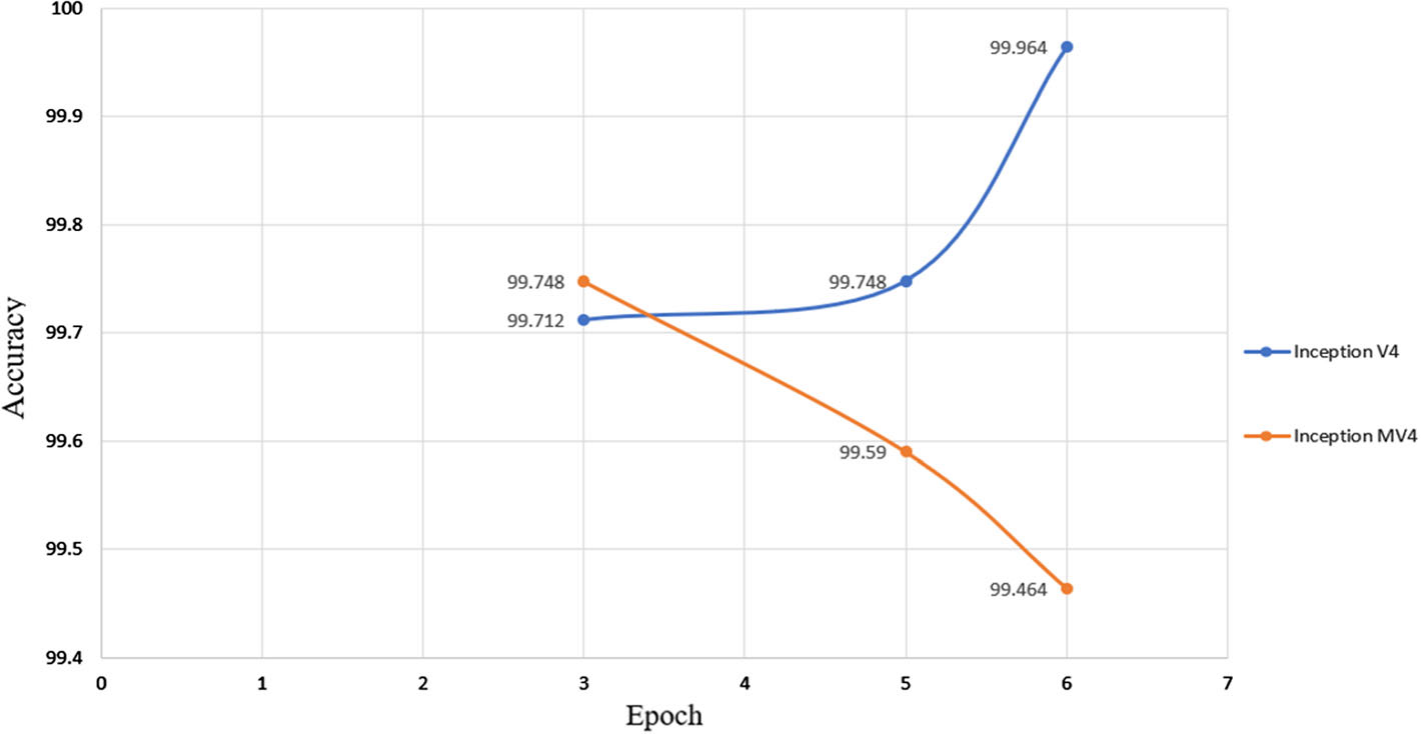
Average accuracy of different epoch for inception V4 and MV4

On the other hand, Table 5 shows the results when the color images were converted into gray images and used in inception V4 model. The network did not reach high accuracy levels in all stages of training. Specifically, the results indicate that the highest accuracy of 72.24% was achieved with SGDM optimization, a learning rate of $1\times {10}^{-4}$ and epoch 30 (Table 6), (Figs. 6, 7 and 8).

**Table 5:** Detection accuracy of inception V4 by using grayscale image

Inception V4 (Grayscale)									
Epoch	SGDM			ADAM			RMSPROP		
	0.001	0.0001	0.0005	0.001	0.0001	0.0005	0.001	0.0001	0.0005
4	54.09	60.5	45.37	61.57	52.31	54.09	46.44	60.85	43.95
5	49.82	54.98	60.32	60.5	45.91	54.09	48.93	46.8	54.09
6	51.96	54.63	54.8	54.09	58.84	54.09	45.91	54.09	54.09
7	57.65	56.58	49.82	48.93	69.93	54.09	54.09	54.09	47.69
8	46.98	66.37	54.09	45.91	54.8	55.87	62.63	55.34	45.91
9	54.09	66.01	48.4	54.09	48.4	54.09	54.09	54.09	50.36
10	52.14	58.54	62.81	55.87	54.8	62.81	54.09	46.09	54.09
20	48.22	63.7	67.62	54.09	49.47	54.09	45.91	62.81	54.09
30	45.91	72.24	62.63	54.09	66.55	50.71	54.09	52.67	54.09

**Table 6:** Performance evaluation results

Methods	TP	TN	FP	FN	Accu	Sen	Spe	P	NPV
Inception V3 color-adam	254	304	0	4	99.29	0.984	1.000	1.000	0.987
Inception V3 color- RMSPROP	257	303	1	1	99.64	0.996	0.997	0.996	0.997
Inception V3 color SGDM	199	281	23	59	85.41	0.771	0.924	0.896	0.826
Inception V3 grayscale-ADAM	246	276	28	12	92.88	0.953	0.908	0.898	0.958
Inception V3 grayscale-RMSPROP	258	293	11	0	98.04	1.000	0.964	0.959	1.000
Inception V3 grayscale-SGDM	218	159	145	40	67.08	0.845	0.523	0.601	0.799
Inception V4 color-ADAm	253	304	0	5	99.11	0.981	1.000	1.000	0.984
Inception V4 color-RMSPROP	254	304	0	4	99.29	0.984	1.000	1.000	0.987
Inception V4 color-SGDM	258	304	0	0	100	1.000	1.000	1.000	1.000
Inception V4 color-Grayscale-ADAM	160	91	213	98	44.66	0.620	0.299	0.429	0.481
Inception V4 Grayscale-RMSPROP	0	304	0	258	54.09	0.000	1.000	NA	0.541
Inception V4 Grayscale-SGDM	210	107	197	48	56.41	0.814	0.352	0.516	0.690

Methods	FPR	FNR	LRP	LRN	AUC	EER	F1
Inception V3 color-adam	0.000	0.016	NA	0.016	0.992	0.008	0.992
Inception V3 color- RMSPROP	0.003	0.004	302.822	0.004	0.996	0.004	0.996
Inception V3 color SGDM	0.076	0.229	10.195	0.247	0.848	0.152	0.829
Inception V3 grayscale-ADAM	0.092	0.047	10.352	0.051	0.931	0.069	0.925
Inception V3 grayscale-RMSPROP	0.036	0.000	27.636	0.000	0.982	0.018	0.979
Inception V3 grayscale-SGDM	0.477	0.155	1.772	0.296	0.684	0.316	0.702
Inception V4 color-ADAm	0.000	0.019	NA	0.019	0.990	0.01	0.990
Inception V4 color-RMSPROP	0.000	0.016	NA	0.016	0.992	0.008	0.992
Inception V4 color-SGDM	0.000	0.000	NA	0.000	1.000	0.000	1.000
Inception V4 color-Grayscale-ADAM	0.701	0.380	0.885	1.269	0.460	0.540	0.507
Inception V4 Grayscale-RMSPROP	0.000	1.000	NA	1.000	0.500	0.500	NA
Inception V4 Grayscale-SGDM	0.648	0.186	1.256	0.529	0.583	0.417	0.632

In general, inception V4 model is distinguished by analyzing color thermal images with high accuracy compared to grayscale thermal images, when the learning rate is $1\times {10}^{-4}$ is used with ADAM optimization. The fifth experiment was conducted for the Modified inception MV4 model. The results of these experiments indicated that the 100% accuracy rate is when the learning rate was $1\times {10}^{-4}$ with SGDM optimization for color images. Table 7 compares the results achieved by inception V4 and inception MV4. The results indicate that the two models achieve a high accuracy of 100%. However, the proposed inception MV4 model is faster than inception V4 by 7%. This result is a key factor in saving time and consumed energy. The use of inception MV4 model contributes significantly to saving energy consumed for arithmetic operations and to the fluidity in arithmetic operations performed by the graphic processor. The MV4 results also suggest that increasing the number of layers in the DCNN model may not necessarily be useful in improving the performance.

**Table 7:** Detection Accuracy and Time Consumption in inception MV4

Inception MV4 color						
Epoch	SGDM		ADAM		RMSPROP	
	0.0001	Time (min)	0.0001	Time (min)	0.0001	Time (min)
3	100	8.9	99.29	10.72	99.29	10.53
4	99.11	11.7	99.47	15.72	95.37	12.8
5	100	13.72	99.64	20.1	99.82	14.35
6	99.11	16.18	96.08	23.45	73.8	19.23
7	100	18.07	98.22	26.87	88.41	23.62
8	99.11	22.8	99.47	32.17	96.26	24.17
9	100	24.53	98.93	39.72	99.47	25.43
10	100	28.03	95.15	50.13	98.22	28.2
20	100	55.57	96.43	114.42	99.64	55.73
30	99.29	81.38	96.79	145.27	91.09	83.33

In this study, a total 1874 thermal images were utilized from DMR-IR database. To investigate the robustness of the model, we modified augmentation algorithm by randomly flipping the training images along the vertical axis, randomly translate them up to 30 pixels, and scale them up to 10% horizontally and vertically. Inception V3 and inception V4 were used with setting to ‘ADAM’ and ‘SGDM’ optimization methods, respectively, and a learning rate of 1e − 4. Then, set inception V3 in classification unit to (Global Average Pooling, Full Connected layer (2048) and SoftMax), but we set in inception V4 in classification unit to (Global Average Pooling + Dropout (0.8) + Full Connected layer (1536) + SoftMax. Besides, the first 10 convolution layers were frozen for both models. The inception V3 results produced an accuracy level of 98.104% with marginal error of ± 1.52%, i.e., deducted from several rounds of repetitions and statistical analysis of results to prove the robustness of the model. Inception V4 achieved an average accuracy of 99.712% with marginal error ± 0.27%. However, inception MV4 has the same settings of inception V4 but showed different results, such as average accuracy of 99.748% with marginal error of ± 0.18%.

**Fig. 9:**
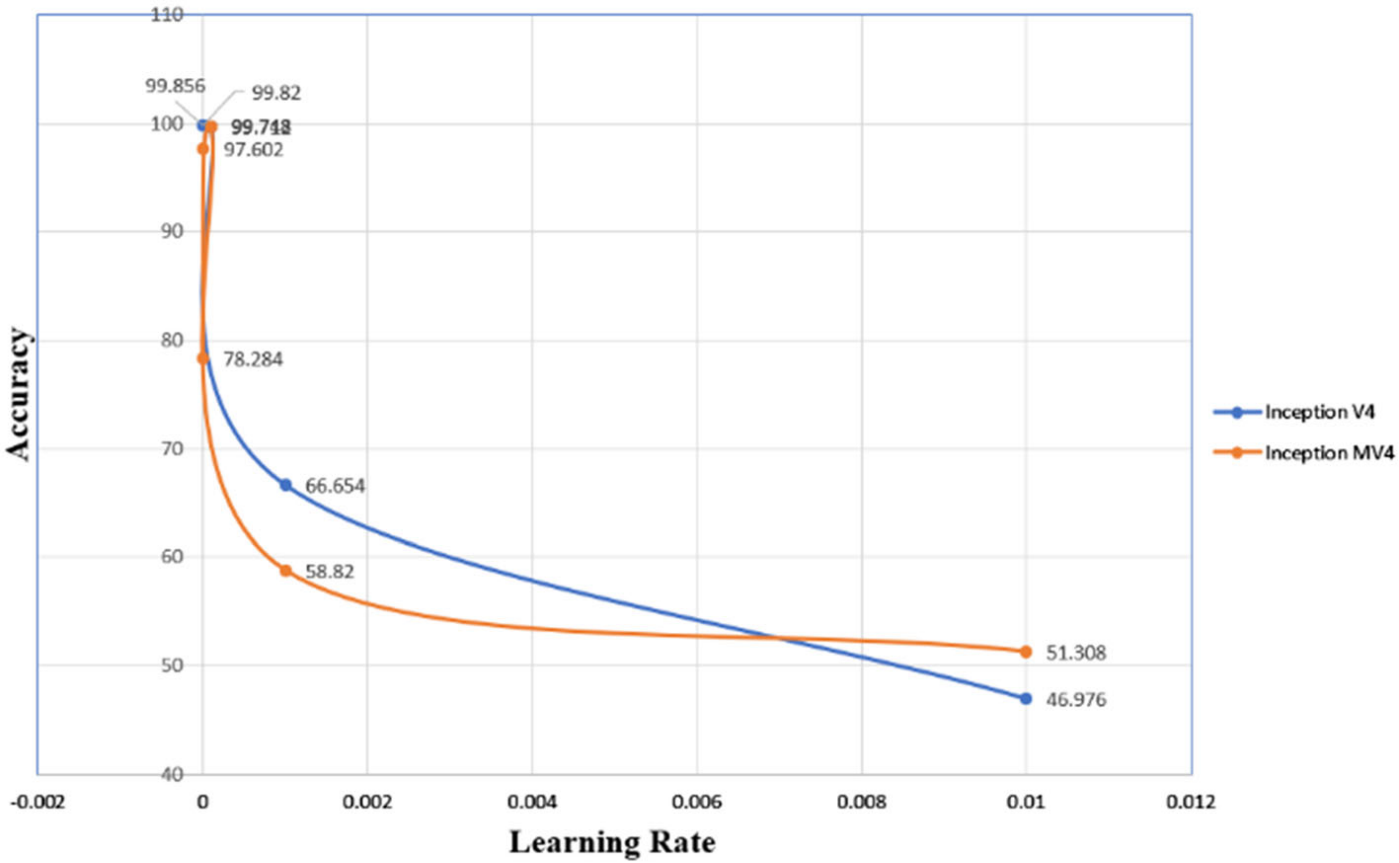
Average accuracy of different learning rate for inception V4 and MV4

Another attempt was made to further fine tune the results, namely changing the learning rate for the optimization method. As can be seen in Fig. 9, the optimal value was somehow close to 1*e*–4, which is the default learning rate for inception MV4, but optimal learning rate for inception V4 was 1*e*–6. Moreover, to emphasize the model robustness, experiments were repeated several times in 3 epochs with setting of networks SGDM optimization method and with using 70% of database for training and 30% for testing. The best overall obtained accuracy for deep convolutional neural network inception V4 and inception MV4 was 99.712% with error ± 0.27% and 99.748% with error ± 0.18%, respectively. Moreover, it showed a very high-speed training in inception V4 and inception MV4 of 9.554 min with error ${}_{-}{}^{+}0.145$ minutes and 7.704 min with error ${}_{-}{}^{+}0.01$ mins of run times, respectively.

In another attempt of fine tuning the proposed framework, we changed the dataset for training to 80, 70, 60, 50 and 40%, and for testing 20, 30, 40, 50 and 60%, respectively. As shown in Fig. 3, inception MV4 achieved its best results with partitioning of 70% for training and 30% for testing. On the other hand, the best results were achieved for inception V4 were 60% for training and 40% for testing dataset partitioning. Furthermore, our MV4 results confirm that increasing the number of layers does not necessarily increase the accuracy, as already been highlighted in [13, 22, 23]. However, generalizing the conclusion for larger sample set than the one used in this study and those in [13, 22, 23] remains to be confirmed in future works. We compared state-of-the-art pre-trained CNN transfer learning models of inception V3 and inception MV4 for performance measurement. However, there are different external factors that affect to accuracy of inception V4 and inception MV4, albeit marginally, like Hardware (RAM, Processor, HDD, and GPU) software, Tools (MATLAB, Python), and versions for detecting breast cancer in early stage. Moreover, to further emphasize the model robustness, we repeated the running of the trained inception V4 and inception MV4 experiments several times with different scenarios by either after shutting down the laptop, shutting the MATLAB application or clear all codes from MATLAB using “clc” and “clear all” commands. The resulting varied random seed indicated slight difference in achieved accuracy levels for inception V4 and inception MV4, with 99.712% with marginal error of ± 0.27% and 99.748% with marginal error of ± 0.18%, respectively.

Table 8 shows the input configurations, number of parameters, learning rate, optimization method used were presented against the output accuracy, error and training time epochs. The 100% accuracy reported by DenseNet and ResNet101 in [5] is hard to verify as no error rate was reported and no reference to model robustness, through image augmentation and repetition of experiments, was discussed. However, the response time taken to achieve the reported accuracy was 70.9 min for DenseNet and 26.4 min for ResNet, respectively, after 10 epochs. Our model achieves the approximately 100% accuracy within a 9.554 min response time for inception V4 and 7.704 min for MV4 utilizing 3 epochs only. VGG16 [3] and CNN-Hyp [2] achieved accuracy levels of 91.18% and 92%, respectively. However, details on error rate, training time epochs, repetition of training, image augmentation and dropout layer fine tuning configuration were not presented also in the paper. On the other hand, inception V3 model used in [25] and our inception V3 model in this paper have used the same ADAM optimization method and learning rate of 1 × 10^–4^, but there are significant different settings such as image augmentation, dropout layer fine tuning configuration, number of parameters used, database sample size and implementation software, as shown in the table. Our inception V3 model produced an accuracy of 98.104% with marginal error of 1.52% compared with the inception V3 model presented in [25] which produced an accuracy of 95.9%, with no reported error rate or training time epochs. Finally, the table shows also that the obtained average accuracy for inception V4 and inception MV4 models were 99.712% with error rate 0.27% and 99.748% with error rate 0.18%, respectively. Furthermore, image augmentation routine and repetition of experiments added to the reliability of the presented results and model robustness while the benchmarking Table 8 demonstrates the novelty of the models and advantages of the proposed work (Tables 9 and 10).

**Table 8:** Benchmarking inception V3, V4, MV4 vs. [2, 3, 5, 20] and [25] (NG = Not Given)

Configuration	CNN-Hyp [2]	VGG-16 [3]	DenseNet [5]	ResNet101 [5]	CNN [20]
Parameters of Augmentation	Horizontal or vertical flip 0–45◦ image rotation 20% zoom normalized noises	Resized to a fixed size (224 × 224 or 227 × 227 pixels)	NG	NG	NG
Configuration	Flatten or global average pooling (GAP) layer, two-unit dense layer with SoftMax	NG	Layer parameter of WeightLearnRateFactor = 10, BiasLearnRateFactor = 10, minibatch size = 10	Layer parameter of WeightLearnRateFactor = 10, BiasLearnRateFactor = 10, minibatch size = 10	NG
	NG	NG	NG	NG	NG
Number of parameters	10,485,760	NG	NG	NG	NG
Optimization method	Bayesian optimization	NG	SGD	SGD	Bayes optimization
Database	DMR-IR database 1140 thermal images Train 50%, validation 20%, and test 30%	173 images 70% for training and 30% for validation	DMR-IR database 80% for training and 20% for validating	DMR-IR database 80% for training and 20% for validating	DMR-IR dataset 3895 thermal images
Learning rate	NG	1e-4	0.001	0.001	0.029
Software	Python3.7	NG	MATLAB	MATLAB	MATLAB
Accuracy	92%	91.18%	100%	100%	98.95%
Error	NG	NG	NG	NG	0.01
Training Time epoch 3	NG	NG	70.9 min in 10 epochs	26.4 min in 10 epochs	NG
Configuration	VGG 16 [25]	Inception V3 [25]	Inception V3	Inception V4	Inception MV4
Parameters of Augmentation	Rotation range = 5, shear range = 0.03, zoom range = 0.03, horizontal flip = True rotation	Rotation range = 5, shear range = 0.03, zoom range = 0.03, horizontal flip = True rotation	Randomly flip the training images along the vertical axis and randomly translate them up to 30 pixels and scale them up to 10% horizontally and vertically	Randomly flip the training images along the vertical axis and randomly translate them up to 30 pixels and scale them up to 10% horizontally and vertically	randomly flip the training images along the vertical axis and randomly translate them up to 30 pixels and scale them up to 10% horizontally and vertically
Configuration	Global Average Pooling, Full Connected layer (512), Dropout (0.5), SoftMax	Global Average Pooling, Full Connected layer (512), Dropout (0.5), SoftMax	Global Average Pooling + Full Connected Layer (2048) + SoftMax	Global Average Pooling + Dropout (0.8) + Full Connected Layer (1536) + SoftMax	Global Average Pooling + Dropout (0.8) + Full Connected Layer (1536) + SoftMax
	Keep last convolution layer unfrozen	Keep last convolution layer unfrozen	First 10 convolution layers frozen	First 10 convolution layers frozen	First 10 convolution layers frozen
Number of parameters	1,678,131	473,440	21,806,882	156,042,082	128,174,466
Optimization method	ADAM	ADAM	ADAM	SGDM	SGDM
Database	1140 thermal images from DMR-IR	1140 thermal images from DMR-IR	1874 thermal images from DMR-IR (70% training &30% Testing)	1874 thermal images from DMR-IR (70% training &30% Testing)	1874 thermal images from DMR-IR (70% training &30% Testing)
Learning rate	1e-4	1e-4	1e-4	1e-4	1e-4
Software	Python	Python	MATLAB	MATLAB	MATLAB
Accuracy	89.7%	95.9%	Average 98.104%	Average 99.712%	Average 99.748%
Error	NG	NG	${}_{-}{}^{+}1.52$%	${}_{-}{}^{+}0.27\mathrm{\%}$	${}_{-}{}^{+}0.18\mathrm{\%}$
Training Time epoch 3	NG	NG	6.376 min with error ${}_{-}{}^{+}0.015$ minutes	9.554 min with error ${}_{-}{}^{+}0.145$ minutes	7.704 min with error ${}_{-}{}^{+}0.01$ minutes

**Table 9:** Average accuracy of different learning rate for inception V4 and MV4

Learning Rate	Inception V4	Time min	Inception MV4	Time min
${1\times 10}^{-2}$	46.976	9.404	51.308	7.806
${1\times 10}^{-3}$	66.654	9.414	58.82	7.768
${1\times 10}^{-4}$	99.712	9.554	99.748	7.704
${1\times 10}^{-5}$	99.82	9.422	97.602	7.766
${1\times 10}^{-6}$	99.856	9.4	78.284	7.704

**Table 10:** Accuracy of several training of inception V4 and MV4

Epoch	Inception V4		Inception MV4	
	Accuracy %	Time (min)	Accuracy %	Time (min)
3	99.64	9.72	100	7.68
	100	9.43	99.64	7.72
	99.64	9.52	99.64	7.72
	99.82	9.55	99.64	7.7
	99.46	9.55	99.82	7.7
Average	99.712	9.554	99.748	7.704

## Conclusion

In conclusion, after conducting all the 468 experimental trials, the results demonstrated that DCNN inception V4 and Modified inception MV4 models are both characterized by high accuracy. Both are very efficient in detecting breast cancer using color thermal images. However, the DCNN Modified inception MV4 with a learning rate of $1\times {10}^{-4}$ , epoch 3 and SGDM optimization method, is faster than inception V4 by 7% in achieving almost 100% accuracy level. This is a key factor in saving time and energy consumed. For some portable applications (e.g., nodes with limited computing resources), a 7% increase in speed with minimal or no degradation in accuracy level is highly appreciated. However, as the number of training epochs are increased, MV-4 accuracy level drops against inception V4 model. Future works should focus on thermal images collected from databases other than DMR, increasing the number of thermal images used, utilizing different optimization methods, and examining the possibility of using such model on a portable device such as a smartphone. Finally, the results motivate the use of DCNN Modified inception MV4 to aid the physician in detecting and diagnosing early breast cancer with possible applications to lung cancer and probably COVID-19. A promising new approach of digital neuromorphic computing toward artificial general intelligence presented in [27–32] could find applications in the broad area of early detection of cancer in the human body.
